# Decision on the Low Bridge Patent

**Published:** 1897-01

**Authors:** 


### SELECTIONS.

### Decision on the Low Bridge Patent.

Below is given the decision of the UNITED STATES COURT OF APPEALS in the case of the International Tooth-Crown Company, complainant and appellant, versus Allen G. Bennett, respondent and appellee.

This is a case in which an appeal is made from the decision of Judge Wheeler, which reversed a former decision by Judges Wallace and Shipman sustaining the Low patent.

This decision, of reversal, by Judge Wheeler, was made the subject of appeal by the International Tooth-Crown Company, and, as will be seen below, this reversal of Judge Wheeler was fully sustained by the U. S. Circuit Court of Appeals, thus, as it seems, putting to test the question of the validity of this patent. The dental profession of this country owes a large debt to the Dental Protective Association, and especially to Dr. J. N. Crouse, the President, who has given a larger share of his time in carrying on the work of the Association, protecting it, so far as may be, from unjust demands and exactions, endeavoring to correct wrongs wherever they may exist, and are reachable. The profession owes Dr. Crouse a debt it can never fully pay, but his work and efforts are held in grateful remembrance by all those who know them.

### United States Circuit Court of Appeals—Second Circuit.

The International Tooth Crown Company, Complainant and Appellant, versus Allen G. Bennett, Respondent and Appellee.

This is an appeal from a decree of the United States Circuit Court, Eastern District of New York, dismissing the bill. The suit is brought to enjoin infringement of Letters Patent No. 238,940, issued March 15, 1881, to James E. Low for an improvement in dentistry. Suit was heretofore brought by this complainant in the Southern District of New York on the same patent (with others) against one Richmond; the patent was sustained and infringement found (30 Fed. Rep. 775). In the suit at bar the Circuit Court found a prior use anticipating the patentee's invention, and for that reason dismissed the bill (72 Fed. Rep. 169).

*Per curiam*, we do not deem it necessary to add anything to the discussion of the case in the Circuit Court. We concur with the learned judge who tried the cause in the conclusion that the real invention of the patentee was a device (consisting of a band or cap and attachments thereto) for permanently inserting artificial teeth without the use of a plate, and without using the gum as a support to the artificial denture, his device holding the tooth in place with sufficient strength to stand the strain of ordinary mastication by attaching it rigidly to the natural denture. This invention could be put in practice by rigidly attaching the artificial tooth either to a single natural tooth adjoining it on one side, or to two adjoining natural teeth, one on each side.

The specification of the patent sets forth that:

"A band of gold or other suitable metal is first prepared and accurately fitted around the tooth adjacent to the vacant spaces to be supplied with an artificial tooth. This band is firmly secured in place by cement, which effectually excludes water or the fluids of the mouth, and is thus permanently attached to the tooth so that it can not be removed without an operation directly for that purpose. It is sometimes sufficient to prepare one of the adjacent teeth in this way; but generally it is desirable to prepare the adjacent teeth on each side of the vacant space. It will always be advisable to do so if the vacant place is to be occupied with more than one tooth."

The invention is not a bridge with two abutments. A bridge with abutments existed in the prior art. The contribution which Low's patent undertook to make to the art was an improved kind of abutment, and that improvement would be availed of, when the process pointed out in the above quotation was applied to a single tooth. The circumstance that in the first claim the words "bands" and "permanent teeth" are in the plural is not significant. The language is made broad enough to cover the process generally, and for that reason uses the plural. It claims "the herein-described method of inserting and supporting artificial teeth," but certainly no one would contend that because of the use of the words "artificial *teeth*" in the plural the claim would not be infringed by the insertion of a single tooth. If the patent were valid the insertion of a single artificial tooth firmly secured to a band of gold accurately fitted and cemented to a natural tooth adjacent to the vacant space to be filled with such artificial tooth, and wholly supported by its attachment to such adjacent natural tooth, without dependence on the gum beneath said artificial tooth, would be an infringement. If this were done before the application for the patent it would be an anticipation. The evidence that this is what was done in the case of Mrs. Mertz is, to our minds, clear and convincing. The date is established beyond a doubt, and it is equally certain that the artificial tooth thus attached was used for years. We concur, therefore, with the Judge who heard the cause in the Circuit Court, that the so-called "Beardslee-Mertz 1877 Permanent Bridge" is an anticipation of the device of the patent.

Complainant contends that the Beardslee-Mertz device does not anticipate the second claim of the patent. The specification, referring to the artificial block or tooth, says: "The lower surface adjacent to the gum is cut away at the back, and only descends to contact with the gum along its front edge, so as to prevent the appearance of an open space between the artificial teeth and the gum." The second claim reads as follows:

"An artificial tooth cut away at the back, so as not to present any contact with the gum except along its front lower edge, and supported by rigid attachment to one or more adjoining permanent teeth, substantially as and for the purpose set forth."

Complainant's counsel in argument and brief contends that in the Beardslee-Mertz device the artificial tooth is not cut away at the back. The very device, however, which was worn by Mrs. Mertz for years, has been produced, and, while it is not cut away in the back as much as are the devices shown in the drawing of the patent, it is manifestly sloped upwards so as not to bear upon the gums. The extent to which the back is cut away is immaterial so long as the cutting away is sufficient to avoid pressure on the gum and leave the artificial tooth or block supported wholly by attachment to the natural dentition. Complainant's own expert speaking of the Beardslee-Mertz device says:

"If the teeth, that is, the artificial crowns, did not bear upon the gums, and were properly and sufficiently supported by adjacent caps or crowns, and the dates were early enough, I suppose they would meet that portion of the second claim of the patent in suit wherein a single natural tooth is used as a support for an adjacent artificial tooth, without bringing the artificial tooth in contact with the gum."

The date is definitely fixed, and the evidence shows that the artificial crown did not bear upon the gum, and was properly and sufficiently supported by the adjacent cap around the crown of the adjacent tooth. Under the construction which must be given to the patent as indicated above, the Beardslee-Mertz device anticipates the second claim as well as the first.

The decree of the Circuit Court is affirmed with costs.

JUDGES LACOMBE AND TOWNSEND.

